# Postmortem evidence of *Babesia* and *Bartonella* infections in chronically ill suicidal young people

**DOI:** 10.1186/s13071-026-07541-8

**Published:** 2026-07-27

**Authors:** Edward B. Breitschwerdt, Cynthia Robveille, Ricardo G. Maggi, Henry H. Lindner, Brent T. Harris, Emily Kingston

**Affiliations:** 1 Intracellular Pathogens Research Laboratory (IPRL), Comparative Medicine Institute, College of Veterinary Medicine, North Carolina State University, Raleigh, NC USA; 2 Hormone Restoration, 230 West Tioga Street, Suite 5, Tunkhannock, PA USA; 3 POND Brain Bank and Department of Neurology, Georgetown University, Washington, DC 20057 USA

**Keywords:** Adolescents, Babesiosis, Bartonellosis, Digital PCR, DNA sequencing, Encephalitis, Enrichment blood culture, Flea and tick-borne pathogens, Neuropsychiatric illnesses, Suicide

## Abstract

**Background:**

Vector-borne diseases represent an emerging global threat to public health, yet the potential role of flea and tick-borne pathogens as a cause or cofactor in neuropsychiatric illnesses and suicidal ideation remains relatively uninvestigated. Co-infections with vector-borne *Babesia* and *Bartonella* spp., as well as infection with more than one *Babesia* species, may represent a more important cause of neurological and neuropsychiatric illnesses than is currently appreciated.

**Methods:**

Postmortem blood, tissue, and urine samples from six chronically ill youths who ended or wanted to end their lives were tested for molecular evidence of *Babesia, Bartonella*, and *Borrelia* spp. using qPCR, dPCR, and enrichment blood culture.

**Results:**

Infections with *Babesia*, *Bartonella*, or both genera were confirmed by DNA sequencing in five of six subjects. Three different *Babesia* spp. were detected in four subjects, of whom two were infected with more than one *Babesia* sp. All six had equivocal *Bartonella* spp. dPCR + or enrichment culture dPCR + results. *Bartonella henselae* was confirmed by DNA sequencing in two individuals. *Borrelia* dPCR was equivocally positive in one subject but could not be confirmed by DNA sequencing.

**Conclusions:**

These findings support the hypothesis that persistent *Bartonella* and *Babesia* spp. infections may have a causal role in chronic illness and potentially suicidality in young people. More research is needed to determine the prevalence and role of these pathogens in patients with chronic physical and neuropsychiatric symptoms and suicidal ideation.

**Graphical Abstract:**

**Figure:**
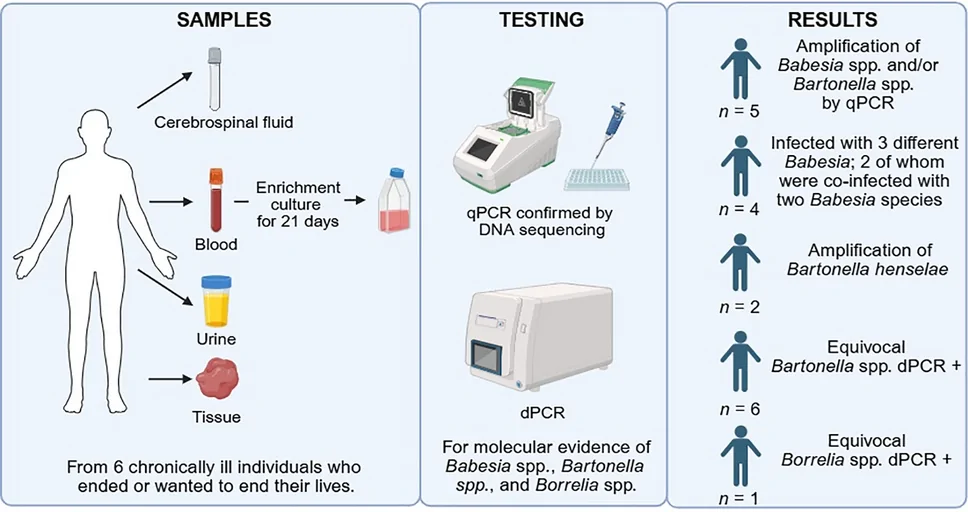

**Supplementary Information:**

The online version contains supplementary material available at https://doi.org/10.1186/s13071-026-07541-8.

## Background

There is growing interest among patients, advocacy groups, physicians, and researchers in the role of microorganisms in chronic neurological and neuropsychiatric illnesses [1–3]. Microbes have been associated with various disorders, including schizophrenia, bipolar disorders, depressive disorders, anxiety disorders, aggressive/violent behavior, and suicidality (see ref [4] for a review). There are individual case reports and small case series, but few comprehensive prospective studies that define the role of specific or polymicrobial infections as cause or cofactor in neuropsychiatric disorders. With the exception of Lyme disease (*Borrelia burgdorferi*), a potential role for flea and tick transmitted, zoonotic vector-borne bacteria or protozoa as a cause of suicidal ideation has been minimally investigated [5, 6].

Prior publications from the Intracellular Pathogens Research Laboratory (IPRL) at North Carolina State University have documented persistent infections with *Babesia* and *Bartonella* spp. in persons with chronic physical, neurological, and neuropsychiatric disorders. *Bartonella* spp. are proteobacteria that are endotheliotropic—their primary niche is the endothelial cell. They enter erythrocytes to disperse throughout the body. *Bartonella* spp. infections can be acquired via animal scratches or bites, fleas, and many arthropods (see ref [7] for a review). *Babesia* spp. are protozoa that are closely related to the *Plasmodium* spp., the cause of human malaria. *Babesia* spp. are highly adapted for tick transmission. In the infected host, *Babesia* merozoites invade erythrocytes, evade elimination by the immune system and coopt erythrocyte nutrition to fulfill metabolic needs (see ref [8] for a review). Both genera constitute stealth pathogens that cause minimal-to-no symptoms in reservoir hosts, but multisystem disease in domestic and wild animals and humans. Clinical outcome is determined by the virulence of the infecting *Babesia* spp. and host’s immune response to the parasites.

The IPRL has documented persistent *Bartonella* spp. infections in persons with neurological and neuropsychiatric disorders, many of whom had extensive arthropod and animal contact [9–15]. In 2019, we reported the case of a boy diagnosed with pediatric acute-onset neuropsychiatric syndrome (PANS) who was homicidal and suicidal [16]. Bartonellosis was first suspected due to unusual cutaneous stretch marks—*Bartonella*-associated cutaneous lesions (BACL) [17, 18]. After *Bartonella henselae* bacteremia was confirmed, he recovered fully with an extended course of antimicrobials. Subsequently, BACL were reported in 29 of 33 persons with neuropsychiatric symptoms who had serological and/or PCR evidence of *Bartonella* spp. exposure and/or infection, 24 of whom reported that the BACL developed in association with their symptoms [19].

Historically, the IPRL has focused on generating improvements in diagnostic testing modalities, improved treatment approaches, and assessment of prevention strategies for zoonotic vector-borne pathogens. Sequential improvements in enrichment blood culture media in conjunction with the more sensitive molecular diagnostic techniques have enhanced *Bartonella*, *Babesia*, and *Borrelia* (*BBB*) spp. detection [20–23]. Following the development and validation of *Bartonella* digital droplet PCR (ddPCR) [20], *Bartonella* DNA was more often amplified from blood or enrichment blood cultures from patients diagnosed with schizophrenia or psychosis compared to controls [24, 25]. Following the subsequent validation of digital PCR (dPCR) tests for *Babesia* and *Borrelia* spp. [22, 23], we documented polymicrobial vector-borne infections in patients reporting neurological and neuropsychiatric symptoms, including suicidality [26–30]. In this study, we report postmortem evidence of *Babesia* and *Bartonella* infections in suicidal young adults and youths.

## Methods

### Case findings

Based upon our publications on neurobartonellosis, the corresponding author was contacted by a parent of each of the six young people in this case cohort requesting testing for postmortem evidence of *BBB* infections. As the subjects had died prior to sample collection, obtaining signed Institutional Review Board consent for research testing was not possible [31]. The Director of the North Carolina State University Institutional Review Board provided a written exemption stating testing of specimens from deceased individuals is NOT Research with Human Subjects as outlined in the (United States) Code of Federal Regulations 45 CFR 46.101 and 45 CFR 46.102. This project does not require IRB review or approval. This is because this project does not meet the definition of a “human subject”: human subject means a living individual about whom an investigator (whether professional or student) conducting research obtains information or biospecimens through intervention or interaction with the individual, and uses, studies, or analyzes the information or biospecimens; or obtains, uses, studies, analyzes, or generates identifiable private information or identifiable biospecimens. As a result, your research does not meet the definition for “human subjects” as noted above because no “living individuals” are involved in the project. The corresponding author obtained signed permission (BioMed Central Consent Form) to publish the case history from a parent of each of the six individuals in this study. Four individuals committed suicide by either drug overdose, hanging or vehicular trauma, one elected assisted suicide after a prolonged, intractable illness, and one was chronically suicidal but died from hemophagocytic syndrome. For each, the parents provided an abbreviated medical history emphasizing symptomatology. Blood, cerebrospinal fluid, urine, or tissues were provided either by the POND Brain Bank, Department of Neurology, Georgetown University, Washington, DC or by the pathologist or mortician who performed the autopsy. Specimens were either refrigerated or frozen prior to overnight shipment to the Intracellular Pathogens Research Laboratory, at the request of the parents. As all individuals resided in different states at the time of death, autopsy interval, postmortem communications, and the number and type of specimens collected varied in each instance. Although delays in tissue collection and storage conditions could negatively affect molecular detection of microbial DNA, these factors would not contribute to enhanced detection.

### Case histories

*Case 1*. A previously healthy and athletic 27-year-old male from the western United States suffered an array of physical and mental symptoms for six years. Prior to illness onset, he had engaged in outdoor activities that exposed him to arthropod vectors-in the western United States, Alaska, Canada, and Switzerland. Symptoms included chronic debilitating fatigue, difficulty remembering, irritability, insomnia, headaches, mental confusion, hallucinations, blurred vision, severe depression, anxiety, panic attacks, shortness of breath, severe chest pain, intermittent bladder dysfunction, constipation and bloating, and poor appetite. He also reported muscle and joint pain with accompanying weakness. During the 1 year of his illness, he lost 50 lbs., which was attributed to dysbiosis. In the last two years of his life, he gained 80 lbs. He was diagnosed with schizoaffective disorder with catatonic psychosis and obsessive–compulsive disorder (OCD). His mental state progressively worsened despite detailed medical evaluations, neuropsychiatric medications, and other interventions provided by numerous medical specialists. He had to withdraw from college. At the onset of illness, his white blood cell count was consistently low, but a bone marrow biopsy showed normal leukocyte maturation. Nineteen months prior to his death, post-infectious autoimmune basal ganglia encephalitis was diagnosed by a positive Cunningham panel (Moleculera biosciences, Oklahoma City, OK). When unusual striae appeared on his skin, bartonellosis was suspected. Serological tests at the Washington State Diagnostic Laboratory and IGeneX Laboratory (Milpitas, CA) indicated exposure to *Bartonella* spp. He was treated for bartonellosis with azithromycin for one month, and then with rifabutin, clarithromycin, and fluconazole for three months. With treatment his neuropsychiatric symptoms began to lessen, but he committed suicide.

Postmortem: DNA sequencing confirmed *Babesia divergens* MO-1, *Babesia microti*, and *Bartonella henselae*. (See testing details below.)

*Case 2*. A 21-year-old female from the southeastern United States experienced a spectrum of neuropsychiatric symptoms for 8 years. Since her early childhood vacationed each summer on Block Island, RI where she had numerous vector exposures including a cat scratch (age unknown). She developed several skin lesions including pyoderma at age 6, an erythematous papular rash at age 7, and a dark, erythematous, 1-cm papule on her lower back at age 13 that was later determined to be a dysplastic nevus. Two years prior to the onset of neuropsychiatric symptoms, she was diagnosed with inflammatory bowel disease and constipation. She developed severe heel and foot pain that persisted for almost a year and was diagnosed as Sever’s disease. Her initial neuropsychiatric symptoms were mood dysregulation, anxiety, depression, memory problems and an inability to focus. Her diagnoses progressed from attention deficit hyperactivity disorder (ADHD) at age 15 to depression, bipolar disorder and schizoaffective disorder. Her symptoms included chronic fatigue, difficulty remembering, irritability, insomnia, poor appetite, mental confusion, hallucinations, anxiety, panic attacks, shortness of breath, and weight loss. Two years prior to death she was diagnosed with psychosis accompanied by mania. After two emergency room visits in June 2022 for delusions and psychosis and two psychiatric hospitalizations, she was transferred to a third psychiatric treatment facility. In August 2022, soon after admission, she committed suicide.

Postmortem: DNA sequencing confirmed *Babesia divergens* MO-1 and *Babesia odocoilei*; dPCR was equivocal (less than 3 positive partitions) for *Bartonella* and *Borrelia* spp. and DNA sequencing attempts were not successful.

**Fig. 1:**
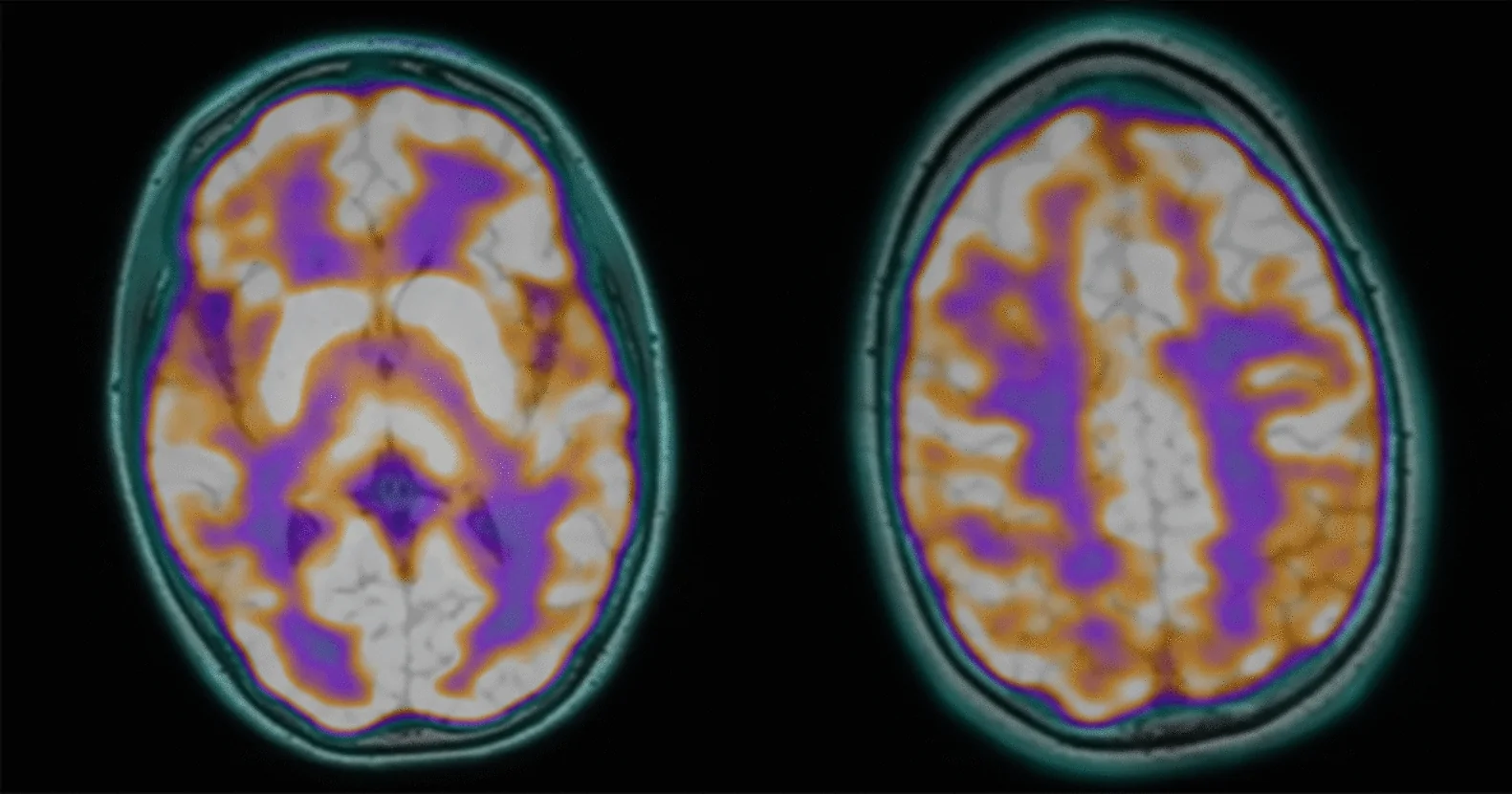
PET/MRI brain scan prior to antibabesial treatment. There is hypermetabolism in the basal ganglia and limbic system (left), indicating inflammation, and hypometabolism in the anterior temporal lobes and parietal lobe (right) indicating neuronal dysfunction. (Lighter areas signify more, darker areas less metabolic activity.)

*Case 3.* A 30-year-old female from the northeastern United States was born to a mother who had exposures to several *Bartonella* spp. vectors. At birth she had a large hemangioma on her shoulder. She was slow to meet developmental milestones. As a child she needed constant distraction and later reported never feeling comfortable or happy. At age 10, her father removed two engorged ticks. She had no rash or fever but slowly became depressed and stopped communicating with friends. By age 13, she was fatigued and developed homicidal and suicidal ideation. A Lyme western Blot assay was negative. At age 18, demands of full-time college caused her symptoms to worsen. She had low mental stamina, developed erythematous papules on her face and back, had frequent urination, numbness in her left lateral thigh, and postural orthostatic tachycardia syndrome (POTS). Extensive testing for toxic, metabolic, and genetic disorders was uninformative. By age 24, she was highly dysphoric, disabled, and was diagnosed clinically with chronic autoimmune encephalitis. Brain magnetic resonance imaging (MRI) revealed white matter hyperintensities. A Mayo Clinic autoimmune encephalitis panel was negative. Serological and molecular tests at IGeneX were negative for *Bartonella*, *Babesia*, *Borrelia*, and *Ehrlichia*. High-dose doxycycline caused fatigue, headache, and flu-like symptoms, but no symptomatic improvements. Atovaquone and azithromycin caused more severe flu-like symptoms with agitation and violent thoughts. These were interpreted as Jarisch–Herxheimer reactions (JHRs)—immune system activation resulting from killing undetermined pathogens. JHRs have been reported with bacterial, fungal, and protozoal infections including malaria [32–34]. Testing at Galaxy Diagnostics (Research Triangle Park, NC) revealed antibodies to *Bartonella henselae* and *Bartonella quintana*. Blood smear fluorescence in situ hybridization (FISH) testing for *Bartonella henselae* 23S rRNA (TLab, Gaithersburg, MD) supported infection. Treatment for bartonellosis with clarithromycin and increasing doses of rifabutin caused weakness, brain fog, irritability, and rage, and no lasting improvements. She failed to respond to various antidepressants, anticonvulsants, antipsychotics, benzodiazepines, ketamine, and other medications and supplements. Only morphine provided relief from her constantly reported “mental pain”. She frequently begged her father to kill her. A Cunningham panel (Molecular biosciences, Oklahoma City, OK) was positive for anti-dopamine receptor D1 antibodies. At age 25, *Babesia* genus 18S rRNA FISH was positive [35] and she was *B. duncani* seroreactive (IGeneX). Again, anti-babesia drugs were not tolerated. Brain positron emission tomography (PET/MRI) revealed marked regional metabolic abnormalities consistent with an inflammatory, autoimmune encephalitis (Fig. 1).

**Fig. 2:**
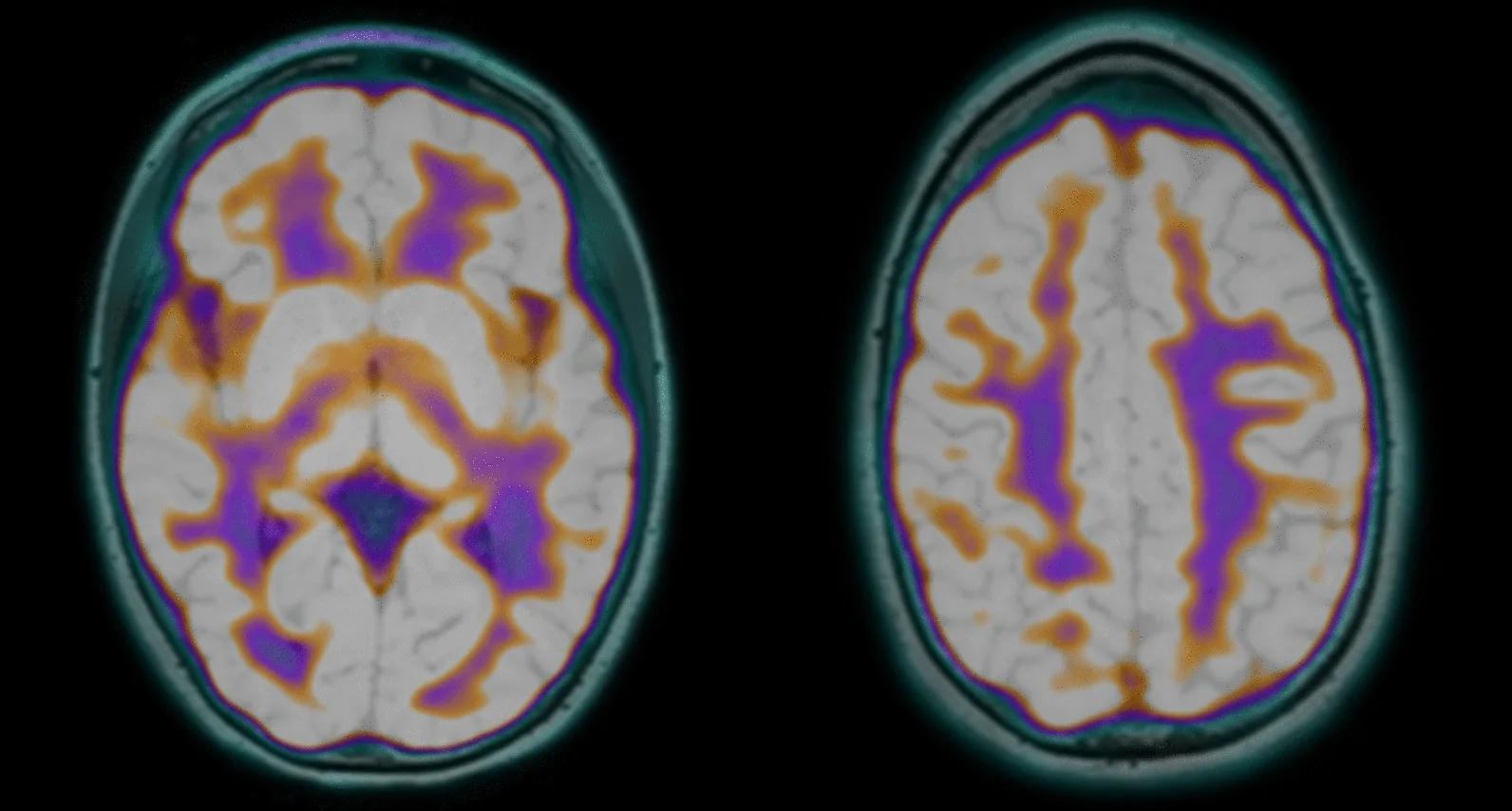
PET/MRI brain scan after 18 months of antibabesial treatment and lumbrokinase showing much improved cortical metabolic activity bilaterally (right) but persisting hyperactivity in the basal ganglia, including the caudate nuclei (left)

A neuroimmunologist prescribed intravenous methylprednisolone for seronegative autoimmune encephalitis, which did not improve symptoms, and during withdrawal she required high corticosteroid doses to eat, sleep, and get out of bed. *Babesia* was not visualized on consecutive blood smears. After beginning plasmapheresis, her father, a physician, administered atovaquone, azithromycin, artesunate, and two 600mg doses of tafenoquine. She experienced extreme fatigue, but within days had dramatic physical and mental improvements accompanied by brisk hemolysis. This antimicrobial regimen, with tafenoquine doses of 600 to 1800 mg weekly, was continued for 6 months during which her symptoms improved despite intermittent JHRs and hemolysis. The potential persistence of *Babesia* parasites despite months of treatment raised questions about the species and its intravascular localization. Her father called the Pennsylvania Tick Lab and learned that they had found *Babesia odocoilei* in 20% of deer ticks. A *Babesia odocoilei* 18S rRNA FISH test (TLab, Gaithersburg, MD) was positive. Due to suspected *B. odocoilei* fibrin sequestration in the microvasculature, oral lumbrokinase (Boluoke^®^), a fibrinolytic enzyme was administered. The following weeks noted improvements, but were accompanied by increasing JHRs, hemolysis, and corticosteroid need. After 5 weeks she developed severe hemolytic anemia, and lumbrokinase was stopped, but her mental and physical functioning remained improved. Over the following 2.5 years, she continued this antibabesial regimen, with rifabutin substituted for azithromycin for greater anti-*Bartonella* activity. At age 28, after 1.5 years of treatment and substantially better mental function, a repeat PET/MRI brain scan revealed markedly improved cortical metabolism but persisting basal ganglia hypermetabolism (Fig. 2).

Repeated courses of lumbrokinase, with the gradual addition of nattokinase, resulted in JHRs and hemolysis. She was able to attend graduate school part-time but continued to assert that she needed to die. While receiving the highest doses for lumbrokinase and nattokinase and requiring high corticosteroid doses, she developed a respiratory infection, was diagnosed with respiratory distress syndrome and a rhinovirus infection. She developed multi-organ system failure and died of hemophagocytic syndrome at age 30.

Postmortem: DNA sequencing confirmed *Bartonella henselae*.

*Case 4*. A 30-year-old female from the Western United States experienced chronic fatigue and a severe gastrointestinal illness of five years duration. Her initial illness started with severe gastrointestinal symptoms in association with travel to Ecuador and a subsequent diagnosis of food poisoning contracted on the trip in May 2018. Due to food intolerance, her weight decreased from 103 to 86 lbs. Sulfur intolerance and Small Intestinal Bacterial Overgrowth (SIBO) were diagnosed after which she regained 12 lbs. Severe fatigue and progressively debilitating foot pain, which substantially limited her mobility, developed within months, requiring a wheelchair for ambulation. Pain progressed to involve her hands and developed wherever pressure was applied: back, shoulders, or head on the pillow. Her severe pain was mitigated after 18 months of exosome infusions. In 2019, she was seroreactive to *B. henselae* and *B. quintana* on two occasions (Galaxy Diagnostics, Research Triangle Park, NC, tests submitted by different physicians). After being diagnosed with bartonellosis, she was treated by multiple physicians with various combination antimicrobial regimens and supportive medications, which resulted in intermittent periods of symptomatic improvement. In September 2021, following a SARS CoV2 vaccination in June and July, her symptoms once again flared; food intolerance with associated weight loss, inability to focus or remember what she was reading, intensified fatigue, and brain fog were reported for the first time. When retested in 2021, she remained *B. quintana* seroreactive, though *Bartonella* alpha proteobacteria enrichment blood culture testing was negative (Galaxy Diagnostics, Research Triangle Park, NC). By September 2023 her weight was 86 lbs and she was considering assisted suicide. Ultimately, due to progressive deterioration in her health, she elected assisted euthanasia in January 2024, weighing 76 lbs. Diagnoses during her illness included Chronic Fatigue Syndrome, Fibromyalgia, Distal Sensory Neuropathy, Mast Cell Activation Syndrome, Long COVID, and Small Intestinal Bacterial Overgrowth (SIBO) that resulted in chronic severe weight loss (approximately 27% of her pre-illness body weight).

Postmortem: dPCR was equivocal (less than 3 positive partitions) for *Bartonella* and *Babesia* spp. and DNA sequencing attempts were not successful.

*Case 5*. A 11-year-old female from the northeastern United States experienced an acute illness of five days duration that was associated with tachycardia, bradycardia, dizziness, fatigue, fever and diarrhea. Prior to illness onset, the child was a “straight A student who thrived socially and academically”. Within three weeks, the prior symptoms recurred, requiring hospitalization for abdominal pain and difficulty swallowing that were attributed to dysautonomia. Following discharge, her pediatrician documented a positive streptococcal antigen test, supporting an additional diagnosis of PANDAS. Two months later, at an autonomic dysfunction clinic, postural orthostatic tachycardia syndrome (POTS) was diagnosed. In the ensuing two and a half years she developed olfactory hallucination, gastroparesis, constipation, diarrhea, anxiety, OCD, aggression/rage, psychosis, emotional lability, separation anxiety, joint hypermobility, striae, increased intracranial pressure, urinary urgency, extreme sensitivity to sound, enthesitis–arthritis (juvenile idiopathic arthritis with inflammation where tendons and ligament attach to bones), arthralgia, periodic loss of vision, auditory hallucinations, and food and drug intolerances. Administration of rituximab and low dose intravenous immunoglobulin (IVIG) for immune-mediated encephalopathy, cefdinir for PANDAS prophylaxis, and IV ceftriaxone infusions and Bactrim (sulfamethoxazole and trimethoprim) for chronic recurrent sinusitis elicited gradual but incomplete improvement over several months of therapy. Following cessation of Bactrim, there was a progressive deterioration in wellbeing with the return of her prior symptoms. Ten months later, at the age of 14, she committed suicide. This was four months after initiating treatment with a selective serotonin reuptake inhibitor, and 12 days after re-institution of Bactrim for bartonellosis (positive blood smear FISH imaging, TLab, Gaithersburg, MD).

Postmortem: DNA sequencing confirmed *Babesia odocoilei*; dPCR was equivocal (less than 3 positive partitions) for *Bartonella* spp and DNA sequencing attempts were not successful.

*Case 6*. A 19-year-old female from the northeastern United States was diagnosed and treated for streptococcal laryngitis. Mononucleosis and streptococcal laryngitis were diagnosed 20 months later in her sophomore year of college. Post-diagnosis, she developed the following: relapsing–remitting, episodic symptoms of social anxiety, obsessive compulsive disorder (OCD), restricted eating, irritability, insomnia, and plaque psoriasis. In addition, her previously excellent academic performance declined. Seven years later, at 26 years old, she was diagnosed with Pediatric Acute Neurological Disorder Associated Streptococcal infection (PANDAS) and was prescribed antibiotics in anticipation of initiating treatments modulating the immune system. Days before treatment initiation, in the midst of a severe flare, she ended her life. Examination of her brain revealed perivascular T-helper lymphocytic infiltrates in blood vessels in the basal ganglia, but not elsewhere in her brain. Detailed clinicopathologic findings for this young woman were previously published [36].

Postmortem: DNA sequencing confirmed *Babesia odocoilei*. dPCR was equivocal (less than 3 positive partitions) for *Bartonella* spp and DNA sequencing attempts were not successful.

### Research testing

Postmortem blood and tissue samples were collected at varying time intervals following death. The number, type and potentially the quality of microbial DNA within specimens available for analysis differed for each individual. Postmortem blood was inoculated into Brugge culture medium supplemented with defibrinated sheep blood (10% by volume) after which DNA was extracted at 7, 14, and 21 days of culture (35⁰C, 100 humidity, 5% CO_2_) [21]. Blood, enrichment blood cultures and biological fluids were extracted using a QIAsymphony SP robot (QIAGEN, Germantown, MD, USA). Manual DNA extraction of approximately 25 mg of tissues was performed using a Qiagen DNeasy^®^ Blood and Tissue kit (Qiagen, Valencia, CA, USA) following the manufacturer’s instructions. The final elution volume was 200 μL.

DNA extracted from each sample was initially screened by qPCR and digital PCR using *Babesia*, *Bartonella* and *Borrelia* spp. primers and probes, as previously described [20, 21]. The volume of template DNA used for molecular testing was 5 μL. Quantitative PCR amplification was performed in an CFX96^®^ (Bio-Rad, Hercules, CA, USA) under the following conditions: 95 °C for 3 min, followed by 45 cycles of denaturing at 94 °C for 15 s, annealing at 68 °C for 15 s, and extension at 72 °C for 15 s. Quantification cycle (Cq) values were obtained by reading fluorescent signals using the FAM (for *Bartonella* DNA detection), HEX (for *Borrelia* DNA detection), and Cy5 (for *Babesia* DNA detection) dye channels. Digital PCR amplification was performed on a QIAcuity One 5plex instrument using 26 k 24-well nanoplates under the following conditions: 95 °C for 2 min, followed by 40 cycles of denaturation at 95 °C for 20 s, and annealing/extension at 64 °C for 20 s. In addition to the previously listed channels, Texas-Red/TAMRA was used for housekeeping gene detection. Results were analyzed using QIAcuity Software Suite version 2.2.0.26. Partitions with fluorescence signals below the defined threshold were classified as negative. To avoid PCR amplicon contamination during sample processing, a one-way workflow was utilized, that included DNA extraction, PCR amplification, and un-inoculated culture controls. Negative controls (for DNA extraction, culture, and PCR reaction) remained negative throughout the study.

Digital PCR results were interpreted as negative (0 positive partitions, i.e., 0 copies/5 µl), equivocal (1 or 2 positive partitions), or positive (3 or more positive partitions, i.e., ≥3 copies/5 µl). All dPCR results were either negative or equivocal. In an effort to generate confirmatory *Babesia* and *Bartonella* DNA sequences, equivocal *Babesia* and *Bartonella* dPCR results were further assessed with SYBR Green-based qPCR assays using genus-specific primers and species-specific primers targeting the *Babesia* Intergenic Spacer region (ITS1 & ITS2) and the *Bartonella henselae* 16S-23S ITS region (Table 1), as previously described [23]. Efforts in our laboratory to confirm *Borrelia* dPCR results by subsequent qPCR amplification and DNA sequencing were not successful.

**Table 1:** Target regions and primers used for *Babesia* and *Bartonella* amplification and detection

Target	Primer name	Primer sequence	Expected size (bp)
*Babesia* genus ITS1	Api18S rRNA-1690s	5′ CTCCTACCGATCGAGTGATCCGGT 3′	⁓ 530–650*
	Api5.8SrRNA-20as	5′ GCTGCGTCCTTCATCGTTGTGTGAG 3′	
*B*. *divergens* ITS1	BdivergenITS1-25s	5′ CTCGGCTTCGACATTTACGTTGTGTAAGCT 3′	⁓ 200
	BdivergenITS1-150as	5′ CAACTACAGTAGTTACACCGYAGTAARCATAC 3′	
*B*. *microti* ITS1	BmicrotiITS1-25s	5′ TATCAGAGTTCTTTGTATCCCATTTGGGTTA 3′	⁓ 285
	BmicrotiITS1-160as	5′ GAAAATACCTTGGGAGTGAGAACGCCCCGT 3′	
*B*. *odocoilei* ITS1	BodocoITS1a-100s	5′ CTGTTGCACTTTTGTGCTTGACGTTGT 3′	⁓ 150
	BodocoITS1a-255as	5′ CAAGCGCAGGGATGGAAACGGA 3′	
*B*. *divergens* ITS2	BdivergensITS2-90s	5′ GACAATCACCTTAATTTCCATAGCATGCTAC 3′	⁓160–170*
	BdivergensITS2-260as	5′ CACTGACAACACTAGCATCATCCACTTG 3′	
*B*. *microti* ITS2	BmicrotiITS2-30s	5′ TCCAAGTTATGCTTTGTGAGTGGTGC 3′	⁓ 400
	BmicrotiITS2-180as	5′ GCGCGGCTAACTAAACTATCTACCACAC 3′	
*B*. *odocoilei* ITS2	BodocoITS2a-65s	5′ CTTTCCTAAAGGTGGCAACCCTTTGCTA 3′	⁓ 220
	BodocoITS2a-290as	5′ AYGCATTAGCCACTTGCCGCAYCACRG 3′	
*Babesia* genus ITS2	Api5.8SrRNA-20s	5′ CTCACACAACGATGAAGGACGCAGC 3′	⁓ 300–555*
	Api28SrRNA-1as	5′ CCGCTGAATTTAAGCATAAAAYTAAGCGG 3′	
*B. divergens* nested ITS1-ITS2	Bdivergens ITS1-25s (outer)	5′ CTCGGCTTCGACATTTACGTTGTGTAAGCT 3′	⁓ 805
	Bdivergens ITS2-260as (outer)	5′ CACTGACAACACTAGCATCATCCACTTG 3′	
	Bdivergens ITS1-70s (inner)	5′ CTTTCTGTGGTTTCGTATTTGC 3′	⁓ 725
	Bdivergens ITS2-210as (inner)	5′ TACAATAGCAATTCTTAGTCGCA 3′	
*Bartonella henselae* ITS	Bh ITS-46s	5′ AGTCCACCGTGGGCTTTGAAAAACGCT 3′	⁓ 170
	Bart ITS-543as	5′ AATTGGTGGGCCTGGGAGGACTTG 3′	

^*^ Expected size is dependent on species and/or strain

## Results

### Symptomatology

All individuals reported neuropsychiatric symptoms. Other symptoms summarized in Table 2 were extracted from the medical histories provided by the parents. There was variation among individuals in this cohort in the number of other organ systems that were reportedly involved. Cases 1, 2, 3, and 5 reported involvements of most major organ systems.

**Table 2:** A summary of major organ system involvement reported in six individuals with suicidal ideation

	Case 1	Case 2	Case 3	Case 4	Case 5	Case 6
Circulatory			X		X	
Digestive	X	X	X	X	X	
Integumentary (skin)	X	X	X		X	X
Musculo-skeletal	X	X	X	X	X	
Nervous	X	X	X	X	X	X
Respiratory	X	X				X
Urinary	X		X		X	

### Molecular microbiology

Samples tested from the six subjects included blood (*n* = 13), enrichment blood culture (*n* = 36), frozen tissues (*n* = 71), and body fluids (*n* = 5) including urine, vitreous, CSF, and a splenic aspirate sample. All 125 DNA extractions from blood, enrichment blood culture, tissues, and body fluids were qPCR negative for *BBB* genus amplification. In contrast, dPCR suggested the presence of *Bartonella*, *Babesia*, or *Borrelia* DNA in various samples from all six individuals. Based upon *BBB* dPCR testing, including blood from case 1, urine from case 2, thyroid tissue from case 4, and enrichment blood cultures from cases 3, 5, and 6, all six subjects had equivocal *Bartonella* results (1–2 positive partitions). Cases 2 and 4 were equivocally *Babesia* dPCR + (1 positive partition) from urine and kidney tissue, respectively. Case 2 generated the only *Borrelia* dPCR (1 positive partition) result from the 125 DNA extractions.

*Babesia* and *Bartonella* PCR results, for which DNA sequence confirmation was successful, are provided in Table 3. Using *B. henselae* species-specific primers, DNA sequence confirmation was obtained for two cases. *Bartonella henselae* DNA was amplified and sequenced from enrichment blood cultures from cases 1 and 3, after incubation for 7 or 14 days, respectively. Using *Babesia* ITS1 and ITS2 primers, infection with *Babesia divergens* MO-1 was confirmed by DNA sequencing in two individuals, case 1 co-infected with *Babesia microti* and case 2 co-infected with *Babesia odocoilei. Babesia odocoilei* DNA was amplified from the 14-day enrichment blood culture from case 5, and from a 7-day enrichment blood culture from case 6.

**Table 3:** Patient designation, PCR positive sample type, *Bartonella* and *Babesia* ITS DNA sequence results following enrichment blood culture and urine DNA extractions

Case	Sample type	Primers used	Species	% Homology (bp)	Compared to GenBank sequence accession
1	Blood culture (C14)	*B*. *divergens* nested ITS1-ITS2	*B*. *divergens* MO-1	99.9 (726/727)	PQ184854
	Blood culture (C14)	*B*. *microti* ITS1	*B*.* microti*	100 (184/184)	SLN871598
	Blood culture (C7)	*Bartonella henselae* ITS	*Bartonella henselae* SA2	100 (55/55)	AF369529
2	Blood Culture (C7)	*Babesia* genus ITS1	*B*. *divergens* MO-1	99.2 (603/608)	PQ184854
		*Babesia* genus ITS2	*B*. *divergens* MO-1	99.8 (417/418)	
		*B. divergens* nested ITS1-ITS2	*B*. *divergens* MO-1	99.9 (686/687)	
	Blood Culture (C21)	*B*. *odocoilei* ITS1	*B*.* odocoilei*	99.1 (108/109)	AY339759
		*B*. *odocoilei* ITS2	*B*.* odocoilei*	94.1 (169/179)	
	Urine	*Babesia* genus ITS1	*B*. *divergens* MO-1	99.8 (434/435)	PQ184854
3	Blood Culture (C14)	*Bartonella henselae* ITS	*Bartonella henselae* SA2	98.1 (104/106)	AF369529
5	Blood Culture (C14)	*Babesia* genus ITS1	*B*.* odocoilei*	99.8 (461/462)	PP693409
6	Blood Culture (C7)	*B*. *odocoilei* ITS1	*B*.* odocoilei*	100 (105/105)	PP693409

## Discussion

Infections with *Babesia*, *Bartonella*, or both genera were confirmed by DNA sequencing in 5 of 6 individuals who committed suicide or reported suicidal ideation. The tragic suffering and deaths of these six young people, ranging in age from 14 to 30 years, clearly define the limitations of contemporary medicine. Medicine is “the science and art dealing with the maintenance of health and the prevention, alleviation, or cure of disease” [37]. Their health was not maintained, and their disease(s) was not alleviated or cured. Given their numerous specialist consultations, diagnostic procedures, and treatment regimens, each clearly wanted to regain their health. The parents, all of whom were professionals, provided consistent access to the highest quality medical care available in the United States, yet were still looking for answers after the death of their children. Four successfully committed suicide and one ended her life with medical aid in dying (MAID). Case 3 was chronically suicidal, but died of hemophagocytic syndrome (hemophagocytic lymphohistiocytosis), an extreme immune system activation disorder that is frequently fatal [38]. Hemophagocytic syndrome has been reported in association with many infections, including *Babesia*, *Bartonella,* and rhinovirus [39–41], which were implicated in this person. The extent to which one or more of these vector-borne parasites contributed to the illnesses or deaths in these young people, or are contributing to neuropsychiatric illnesses worldwide, needs to be explored. In addition to neuropsychiatric symptoms, it is noteworthy that these individuals experienced other medical problems that have been reported in association with vector-borne parasite infections, including severe fatigue, joint pain, foot pain, substantial fluctuations in body weight, dysbiosis, dysautonomia, and SIBO. These observations support the hypothesis that the patients' syndromes may have stemmed from physiological issues, rather than solely mental or psychosocial abnormalities.

Due to the proven chronicity of *Babesia* and *Bartonella* spp. infections, it is not possible to determine the mode of transmission, when transmission occurred, the duration of infection prior to the development of symptoms, or the overall duration of infection prior to death. Exposures to animal reservoirs or arthropod vectors that transmit *Babesia* and *Bartonella* spp. were either reported or likely in all cases. *Babesia* species are tick transmitted, whereas *Bartonella henselae* is primarily transmitted by cat scratches or bites and by *Ctenocephalides felis.* Case 3’s history suggested possible intrauterine or perinatal *Bartonella* infection, which has been reported [42, 43]. Subsequent or sequential engorged tick attachments may have resulted in *Bartonella* and *Babesia* spp. coinfection. Coinfection, as well as infection with more than one *Babesia* sp., may increase the risk of neurological and neuropsychiatric illnesses [15, 16, 19, 24–30]. Neurological symptoms have most often been reported with acute human babesiosis [44]. In 37 children with pediatric bipolar disorder, laboratory tests supported a diagnosis of babesiosis in 51% and bartonellosis in 49% [45]. There is increasing evidence that some psychiatric disorders are mild forms of infection-related encephalitis [2, 4]. As intravascular pathogens, *Babesia* and *Bartonella* spp. circulate through blood vessels in the brain where they could trigger inflammation in surrounding brain tissue. Basal ganglia, subcortical structures that regulate emotions, motivation, and motor activity, are particularly sensitive to inflammatory cytokines [46], which reduce dopamine signaling, causing fatigue, depression, and anhedonia [47]. Cases 1, 3, 5, and 6 had evidence of basal ganglia encephalitis (BGE). Cases 1 and 3 had positive Cunningham panels [48], suggesting autoimmune BGE (PANDAS and PANS) [49, 50]. Case 3 had PET scan evidence of hypermetabolism, indicating inflammation, in the basal ganglia and limbic system. Cases 5 and 6 were diagnosed with PANDAS and case 6 had perivascular lymphocytic infiltration involving the basal ganglia. Further research is needed to clarify a potential role for infection in BGE, neuropsychiatric disorders, and suicide.

Based upon DNA sequencing, three different tick-transmitted *Babesia* spp. were documented in four of the six subjects, of whom two were infected with more than one *Babesia* sp. Despite not being diagnosed with or treated for babesiosis, postmortem testing confirmed *Babesia* spp. infections in cases 1, 2, 4 and 6. Clinical research focusing on persistent human *Babesia* spp. infections is limited. Case 1, co-infected with *B. divergens-*MO-1 and *B. microti*, was a hiker who had frequent animal and arthropod vector exposures*.* Recent genome sequencing data indicate that *B. divergens*-MO-1 is distinct from *Babesia divergens,* which in Europe is transmitted by *Ixodes ricinus* and infects cattle and humans [51]. In the US, eastern cottontail rabbits (*Sylvilagus floridanus*) are a reservoir host for *B. divergens*-MO-1. The putative vector is *Ixodes dentatus*, which feeds on rabbits, birds, and infrequently humans [52]. Additional research is needed to define other potential reservoir hosts and competent tick vectors for *B. divergens*-MO-1, an emerging human parasite in North America [28–30].

*Babesia odocoilei* has recently been identified in 20 chronically ill humans [23, 26, 29, 30, 53]. In most cases, several blood specimens, in conjunction with enrichment blood cultures, were tested to generate a positive PCR result. In North America, *B. odocoilei*’s primary host is the white-tailed deer (*Odocoileus virginianus*) [54, 55]. The roe deer in Europe is host for *B. venatorum,* a close relative to *B. odocoilei* that infects humans [56, 57]. Current U.S. guidelines dealing with acute babesiosis, state that *B. microti* is the most frequent cause of human babesiosis, and infection can be diagnosed with a blood smear [58]. These guidelines do not address *B. odocoilei* or management of chronic babesiosis. Case 3 had two engorged adult female *I. scapularis* ticks removed in Pennsylvania. The Pennsylvania Tick Laboratory found *B. odocoilei* in 17.5% of the *I. scapularis* ticks, *B. microti* in < 3%, and no *B. duncani* [59]. Another survey of *I. scapularis* ticks in Pennsylvania found *B. odocoilei* in 15.4% and *B. microti* in 0.7% [60]. *Babesia odocoilei* was found in 14% of *I. scapularis* ticks taken from hunter-harvested white-tailed deer in Wisconsin [61]. A survey of *Ixodes pacificus* (western blacklegged) ticks in California found *B. odocoilei* in 3%, and no *B. microti* or *B. duncani* [62]. Of epidemiological concern is the fact that *B. odocoilei* is transmitted transovarially in *Ixodes* ticks, from an infected female to many of her 1000 or more eggs [63, 64]. Nearly invisible larvae, hatching in the late summer, can transmit *B. odocoilei*. In contrast, *B. microti* [65] and *B. burgdorferi* [66] are not transovarially transmitted; larvae must feed and molt into nymphs to transmit these pathogens.

Case 2, co-infected with *B. divergens-*MO-1 and *B. odocoilei,* vacationed each summer on Block Island, RI, where *B. odocoilei* has been found in *I. scapularis* ticks [67]. Cases 5 and 6, both from the historically endemic *I. scapularis* northeastern United States, were infected with *B. odocoilei*. *Babesia* sequestration in microcapillaries may be a contributor to symptom variability among patients, the establishment of persistent infection and the lack of effectiveness of anti-babesia drugs.

Among professionals, and compared to the general population, veterinary workers have the highest, or among the highest, risk of committing suicide [68, 69]. Veterinary workers also have an increased risk of *Bartonella* spp. infections [70–73] and potentially *Babesia* spp. infections [26]. Co-infections with *Bartonella* spp. and *B. odocoilei* were reported in a veterinarian and in veterinary technicians [26]. In their 2023 comprehensive review involving suicide among veterinarians by DeSilva and colleagues, the words “infection” and “zoonoses” do not appear anywhere in the manuscript, illustrating the fact that zoonotic infections are often not considered among the current investigated risk factors for suicidal ideation [69]. Future longitudinal studies addressing neuropsychiatric illnesses and suicidal risks in veterinary workers should include zoonotic disease assessments.

Most likely due to the small quantity of pathogen DNA in the blood, urine, CSF, and tissues of infected individuals, molecular documentation of *BBB* spp. using qPCR or dPCR continues to lack sensitivity. In this study, no blood, tissue, or CSF sample DNA extraction yielded a positive *BBB* genus qPCR result. Low copy (< 3 copies/5 µl) *Babesia* and *Bartonella* dPCR results provided the initial indication of infection. In all instances, dPCR amplified a very low (1–2 positive partitions) quantity of *BBB* DNA from blood, urine, and tissue samples. Importantly, blood culture facilitated successful DNA sequencing of *Babesia* or *Bartonella* for 5 individuals. Combining enrichment culture of blood or other aseptically obtained diagnostic specimens increases sensitivity of detection for *Bartonella* and *Babesia* spp. [30, 74–77]. In another study, the utility of enrichment culture was illustrated by negative qPCR/dPCR results following tissue DNA extraction, then a positive *B. henselae* result when a brain biopsy was incubated in two enrichment culture media [29]. In this study, only one of 125 DNA extractions yielded a single *Borrelia* positive partition from a postmortem urine sample. After subcutaneous inoculation in mice, *B. burgdorferi* DNA was rapidly cleared from the blood but was PCR-amplified from the urinary bladder [78]. In dogs with hemangiosarcoma, *Bartonella* dPCR was more sensitive than qPCR when testing blood, but was not more sensitive than qPCR when testing tissue from *Bartonella*-infected dogs [79]. Potentially, the larger quantity of host DNA in tissues compared to peripheral blood decreases qPCR and dPCR sensitivity. Additional research into techniques that will facilitate direct detection of antigens or DNA of these fastidious vector-borne pathogens is needed.

Postmortem detection of pathogen DNA does not prove their causative role in the protracted illnesses or deaths of these young people. Other infectious agents, as well as other cofactors or diseases, may have played a role. Due to the fact that blood specimens were obtained postmortem, *Bartonella* enrichment blood culture may have been compromised by postmortem translocation of rapidly growing bacteria [80]. As liquid enrichment cultures were not subcultured onto blood agar plates, the presence of other bacteria was not assessed. Several of the cases had positive tests for parasites in life that we could not confirm during postmortem testing. False negative *BBB* DNA test results may have been caused by the low number of organisms present in the blood and tissues, and by the variability in the number, quality, and type of specimens. Delays of up to several days between the time of death and autopsy specimen collection could have reduced the sensitivity of *BBB* DNA amplification. All subjects had received antimicrobial drugs for varying periods prior to specimen collection, which likely decreased the quantity of parasite DNA in blood and tissue extractions.

## Conclusions

These bacterial and protozoal vector-borne parasites have long evolutionary relationships with one another, their vectors, and their preferred hosts. This co-evolution has facilitated the perpetuation of the parasite, vector, and host. Opportunistic infection of human hosts can result in minimal or no symptoms, acute self-limiting illness, severe life-threatening illness, or chronic indolent illness potentially spanning many years. This case series supports a role for persistent *Babesia* and *Bartonella* spp. infections in very ill suicidal people. As both pathogens can inhabit the microvasculature throughout the body, including the brain and other tissues, chronic low-level inflammation involving emotional centers of the basal ganglia and limbic system (case 6) might induce enough cognitive and emotional dysfunction in conjunction with a lengthy period of physical suffering to provoke suicide. This hypothesis requires further investigation. Postmortem studies will require concerted efforts by the family and medical establishment to obtain optimal specimens for molecular and microscopic examination as soon as possible after death. Hopefully this case cohort will stimulate additional research investigating the role of chronic *Babesia* and *Bartonella* infections in neuropsychiatric illnesses and suicide.

## Supplementary Information

Below is the link to the electronic supplementary material.Supplementary file1 (PNG 748 KB)

## Data Availability

Data supporting the main conclusions of this study are included in the manuscript.

## Abbreviations

IPRL: Intracellular Pathogens Research Laboratory
BACL: *Bartonella*-associated cutaneous lesions
*BBB*: *Bartonella*, *Babesia*,* Borrelia*
BGE: Basal ganglia encephalitis
CSF: Cerebrospinal fluid
PCR: Polymerase chain reaction
dPCR: Digital PCR
ddPCR: Digital droplet PCR
qPCR: Quantitative PCR
JHR: Jarisch–Herxheimer reaction
OCD: Obsessive–compulsive disorder
POTS: Postural orthostatic tachycardia syndrome
